# Genome Mining Coupled with OSMAC-Based Cultivation Reveal Differential Production of Surugamide A by the Marine Sponge Isolate *Streptomyces* sp. SM17 When Compared to Its Terrestrial Relative *S. albidoflavus* J1074

**DOI:** 10.3390/microorganisms7100394

**Published:** 2019-09-26

**Authors:** Eduardo L. Almeida, Navdeep Kaur, Laurence K. Jennings, Andrés Felipe Carrillo Rincón, Stephen A. Jackson, Olivier P. Thomas, Alan D.W. Dobson

**Affiliations:** 1 School of Microbiology, University College Cork, T12 YN60 Cork, Ireland; e.leaodealmeida@umail.ucc.ie (E.L.A.); andres-felipe.carrillo@alumnos.unican.es (A.F.C.R.); stevejackson71@hotmail.com (S.A.J.); 2 Marine Biodiscovery, School of Chemistry and Ryan Institute, National University of Ireland Galway (NUI Galway), University Road, H91 TK33 Galway, Ireland; navdeep.kaur@nuigalway.ie (N.K.); laurence.jennings@nuigalway.ie (L.K.J.); olivier.thomas@nuigalway.ie (O.P.T.); 3 Environmental Research Institute, University College Cork, T23 XE10 Cork, Ireland

**Keywords:** genome mining, OSMAC, phylogenomics, secondary metabolites, surugamides, surugamide A, marine sponge-associated bacteria, *Streptomyces*, *albidoflavus* phylogroup

## Abstract

Much recent interest has arisen in investigating *Streptomyces* isolates derived from the marine environment in the search for new bioactive compounds, particularly those found in association with marine invertebrates, such as sponges. Among these new compounds recently identified from marine *Streptomyces* isolates are the octapeptidic surugamides, which have been shown to possess anticancer and antifungal activities. By employing genome mining followed by an one strain many compounds (OSMAC)-based approach, we have identified the previously unreported capability of a marine sponge-derived isolate, namely *Streptomyces* sp. SM17, to produce surugamide A. Phylogenomics analyses provided novel insights on the distribution and conservation of the surugamides biosynthetic gene cluster (*sur* BGC) and suggested a closer relatedness between marine-derived *sur* BGCs than their terrestrially derived counterparts. Subsequent analysis showed differential production of surugamide A when comparing the closely related marine and terrestrial isolates, namely *Streptomyces* sp. SM17 and *Streptomyces albidoflavus* J1074. SM17 produced higher levels of surugamide A than *S. albidoflavus* J1074 under all conditions tested, and in particular producing >13-fold higher levels when grown in YD and 3-fold higher levels in SYP-NaCl medium. In addition, surugamide A production was repressed in TSB and YD medium, suggesting that carbon catabolite repression (CCR) may influence the production of surugamides in these strains.

## 1. Introduction

Members of the *Streptomyces* genus are widely known to be prolific producers of natural products. Many of these compounds have found widespread use in the pharmaceutical industry as antibiotics, immunosuppressant, antifungal, anticancer, and anti-parasitic drugs [1]. However, there continues to be an urgent need to discover new bioactive compounds, and especially antibiotics, primarily due to the emergence of antibiotic resistance in clinically important bacterial pathogens [2,3]. In particular, the increase in multi-resistant ESKAPE pathogens (*Enterococcus faecium*, *Staphylococcus aureus*, *Klebsiella pneumoniae*, *Acinetobacter baumannii*, *Pseudomonas aeruginosa*, and *Enterobacter* species) has focused research efforts to develop new antibiotics to treat these priority antibiotic-resistant bacteria [4].

Up until relatively recently, marine ecosystems had largely been neglected as a potential source for the discovery of novel bioactive compounds, in comparison to terrestrial environments, primarily due to issues of accessibility [5]. Marine sponges are known to host a variety of different bacteria and fungi, which produce a diverse range of natural products, including compounds with antiviral, antifungal, antiprotozoal, antibacterial, and anticancer activities [5,6]. Marine sponge-associated *Streptomyces* spp. are a particularly important source of bioactive compounds, with examples including *Streptomyces* sp. HB202, isolated from the sponge *Halichondria panicea,* which produces mayamycin, a compound with activity against *Staphylococcus aureus* [7]; and streptophenazines G and K, with activity against *Bacillus subtilis* [8]; together with *Streptomyces* sp. MAPS15, which was isolated from *Spongia officinalis,* which produces 2-pyrrolidine, with activity against *Klebsiella pneumoniae* [9]. Additionally, our group has reported the production of antimycins from *Streptomyces* sp. SM8 isolated from the sponge *Haliclona simulans*, with antifungal and antibacterial activities [10,11]. In further work, we genetically characterised 13 *Streptomyces* spp. that were isolated from both shallow and deep-sea sponges, which displayed antimicrobial activities against a number of clinically relevant bacterial and yeast species [12,13]. Amongst these strains, the *Streptomyces* sp. SM17 demonstrated an ability to inhibit the growth of *E. coli* NCIMB 12210, methicillin-resistant *S. aureus* (MRSA), and *Candida* spp., when employing deferred antagonism assays [12,13].

Among other clinically relevant natural products derived from marine *Streptomyces* isolates are the recently identified surugamides family of molecules. The cyclic octapeptide surugamide A and its derivatives were originally identified in the marine-derived *Streptomyces* sp. JAMM992 [14], and have been shown to belong to a particularly interesting family of compounds due not only to their relevant bioactivity, but also due to their unusual metabolic pathway involving D-amino acids [14,15,16]. Since their discovery, concerted efforts have been employed in order to chemically characterise these compounds and determine the genetic mechanisms involved in their production [14,15,17,18,19,20]. The surugamides and their derivatives have been shown to possess a number of bioactivities, with the surugamides A–E and the surugamides G–J being shown to possess anticancer activity by inhibiting bovine cathepsin B, a cysteine protease reported to be involved in the invasion of metastatic tumour cells [14,16]; while another derivative, namely acyl-surugamide A, has been shown to possess anti-fungal activity [16]. It has been determined that the non-ribosomal peptide synthase-encoding *surABCD* genes are the main biosynthetic genes involved in the biosynthesis of surugamides and their derivatives [19], with these genes being involved in the production of at least 20 different compounds [16]. Surugamides A–E have been reported to be produced by the *surA* and *surD* genes, while the linear decapeptide surugamide F has been shown to be produced by the *surB* and *surC* genes, involving a unique pattern of intercalation of the biosynthetic genes [19]. Further metabolic pathways studies have reported that the expression of the *surABCD* gene cluster is strongly regulated by the *surR* transcriptional repressor [16], while the cyclisation of the cyclic surugamides has been shown to involve a penicillin binding protein (PBP)-like thioesterase encoded by the *surE* gene [17,18,21].

Although apparently widespread in marine-derived *Streptomyces* isolates [18,19], the production of surugamides has also been reported in the *S. albidoflavus* strain J1074 [16,22], a derivative of the soil isolate *S. albus* G [23,24]. The *S. albidoflavus* strain J1074 is a well-characterised *Streptomyces* isolate, which is frequently used as a model for the genus and has commonly been successfully employed in the heterologous expression of biosynthetic gene clusters (BGCs) [25,26,27,28,29]. This strain was originally classified as an *S. albus* isolate, however, due to more recent taxonomy studies, it has been reclassified as a *S. albidoflavus* species isolate [30,31]. Interestingly, surugamides and their derivatives have been shown to only be produced by *S. albidoflavus* J1074 under specific conditions, such as when employing chemical stress elicitors [16], and more recently when cultivating the strain in a soytone-based liquid-based medium SG2 [22].

In a previous study [32], we reported that the *S. albidoflavus* J1074 and *Streptomyces* sp. SM17 possessed morphological and genetic similarities. Differences were observed, however, when both strains were exposed to high salt concentrations using culture media, such as TSB or ISP2, in which the marine sponge-derived strain SM17 grew and differentiated more rapidly in comparison with the soil strain *S. albidoflavus* J1074, which appeared to have trouble growing and differentiating when salts were present in the growth medium [32]. Genome mining based on the prediction of secondary metabolites BGCs also showed many similarities between the two strains [32]. Among these predicted BGCs, both the *S. albidoflavus* J1074 and *Streptomyces* sp. SM17 isolates appeared to possess the *sur* BGC, encoding for the production of surugamides A/D. Due to the fact that marine-derived *Streptomyces* isolates have been shown to produce good levels of surugamides when grown under standard conditions [18,19], and that production of surugamides and derivatives can be induced in the presence of chemical stress elicitors in *S. albidoflavus* J1074 [16], it appears likely that marine-derived *Streptomyces* isolates and their *sur* BGCs could share genetic similarities that might help to optimise production of the compound. To investigate this possibility we 1) employed genome mining approaches together with phylogenomics in order to better characterise the SM17 strain, and to investigate the distribution and differences/similarities between marine- (or aquatic saline-) and terrestrial-derived *sur* BGCs and *sur* BGC-harbouring microorganisms; and 2) experimentally compared the metabolic profiles of surugamide A production between a marine (SM17) and a terrestrial (J1074) *Streptomyces* isolate. With respect to the latter, we employed an "one strain many compounds" (OSMAC)-based approach, which has been shown to be a useful strategy in eliciting production of natural products from silent gene clusters by employing different culture conditions [33,34]; together with analytical chemistry methods such as liquid chromatography–mass spectrometry to monitor production of surugamide A in both *S. albidoflavus* J1074 and *Streptomyces* sp. SM17.

## 2. Materials and Methods

### 2.1. Bacterial Strains and Nucleotide Sequences

The *Streptomyces* sp. SM17 strain was isolated from the marine sponge *Haliclona simulans*, from the Kilkieran Bay, Galway, Ireland, as previously described [13]. The *Streptomyces albidoflavus* J1074 strain was provided by Dr Andriy Luzhetskyy (Helmholtz Institute for Pharmaceutical Research Saarland, Saarbrücken, Germany). Their complete genome sequences are available from the GenBank database [35] under the accession numbers NZ_CP029338 and NC_020990, for *Streptomyces* sp. SM17 and *S. albidoflavus* J1074, respectively. The surugamides biosynthetic gene cluster (*sur* BGC) sequence used as a reference for this study was the one previously described in *Streptomyces albidoflavus* LHW3101 (GenBank accession number: MH070261) [18]. Other genomes used in this study’s analyses were obtained from the GenBank RefSeq database [35].

### 2.2. Phylogenetic Analyses

The NCBI BLASTN tool [36,37] was used to determine the closest 30 *Streptomyces* strains with complete genome available in the GenBank RefSeq database [35] to the *Streptomyces* sp. SM17. Then, phylogeny analysis was performed with the concatenated sequences of the 16S rRNA, and the housekeeping genes *atpD*, *gyrB*, *recA*, *rpoB*, and *trpB*. The sequences were aligned using the MAFFT program [38], and the phylogeny analysis was performed using the MrBayes program [39]. In MrBayes, the general time reversible (GTR) model of nucleotide substitution was used [40], with gamma-distributed rates across sites with a proportion of invariable sites, with 1 million generations sampled every 100 generations. Final consensus phylogenetic tree generated by MrBayes was processed using MEGA X [41], with a posterior probability cut-off of 95%.

Phylogeny analysis of the surugamides biosynthetic gene cluster (*sur* BGC) was performed by using the *S. albidoflavus* LHW3101 *sur* BGC nucleotide sequence as reference [18] and searching for similar sequences on the GenBank RefSeq database using the NCBI BLASTN tool [35,36,37], only taking into account complete genomes. The genome regions with similarity to the *S. albidoflavus* LHW3101 *sur* BGC undergone phylogeny analysis using the same aforementioned tools and parameters.

### 2.3. Prediction of Secondary Metabolites Biosynthetic Gene Clusters

In order to assess the similarities and differences between the *Streptomyces* isolates belonging to the *albidoflavus* phylogroup, in regard to their potential to produce secondary metabolites, BGCs were predicted in their genomes, using the antiSMASH (version 5 available at https://docs.antismash.secondarymetabolites.org/) program [42]. The predicted BGCs were then processed using the BiG-SCAPE program (version 20190604, available at https://git.wageningenur.nl/medema-group/BiG-SCAPE) [43], with the MiBIG database (version 1.4 available at https://mibig.secondarymetabolites.org/) as reference [44], and similarity clustering of gene cluster families (GCFs) was performed. The similarity network was processed using Cytoscape (version 3.7.1, available at https://cytoscape.org/) [45].

### 2.4. Gene Synteny Analysis

The genome regions previously determined to share similarities with the *S. albidoflavus* LHW3101 *sur* BGC were manually annotated, for the known main biosynthetic genes (*surABCD*), the penicillin binding protein (PBP)-like peptide cyclase and hydrolase *surE* gene, and the gene with regulatory function *surR* [15,16,17,18,19,21]. This was performed using the UniPro UGENE toolkit (version 1.32.0, available at http://ugene.net/) [46], the GenBank database, and the NCBI BLASTN tool (available at https://blast.ncbi.nlm.nih.gov/Blast.cgi, accessed on June 2019) [35,36,37]. The gene synteny and reading frame analysis was performed using the UniPro UGENE toolkit [46] and the Artemis genome browser (version 18.0.0, available at https://www.sanger.ac.uk/science/tools/artemis) [47].

### 2.5. Diagrams and Figures

All the Venn diagrams presented in this study were generated using the Venn package in R [48,49], and RStudio [50]. All the images presented in this study were edited using the Inkscape program (available from https://inkscape.org/).

### 2.6. Strains Culture, Maintenance, and Secondary Metabolites Production

The same culture media and protocols were employed for both isolates *Streptomyces* sp. SM17 and *Streptomyces albidoflavus* J1074. Glycerol stocks were prepared from spores collected from soya-mannitol (SM) medium after 8 days of cultivation at 28 °C and preserved at −20 °C. To verify the secondary metabolites production profile, spores were cultivated for 7 days on SM agar medium at 28 °C, then pre-inoculated in 5 mL TSB medium, and cultivated at 28 °C and 220 rpm for 2 days. Then, 10% (*v*/*v*) of the pre-inoculum was transferred to 30 mL of the following media: TSB; SYP-NaCl (1% starch, 0.4% yeast extract, 0.2% peptone, and 0.1% NaCl); YD (0.4% yeast extract, 1% malt extract, and 4% dextrin pH 7.0); P1 (2% glucose, 1% soluble starch, 0.1% meat extract, 0.4% yeast extract, 2.5% soy flour, 0.2% NaCl, and 0.005% K_2_HPO_4_ pH 7.3); P2 (1% glucose, 0.6% glycerol, 0.1% yeast extract, 0.2% malt extract, 0.6% MgCl_2_.6H_2_O, 0.03% CaCO_3_, and 10% sea water); P3 (2.5% soy flour, 0.75% starch, 2.25% glucose, 0.35% yeast extract, 0.05% ZnSO_4_ × 7H_2_O, and 0.6% CaCO_3_ pH 6.0); CH-F2 (2% soy flour, 0.5% yeast extract, 0.2% CaCO_3_, 0.05% citric acid, 5% glucose, and pH 7.0); SY (2.5% soluble starch, 1.5% soy flour, 0.2% yeast extract, and 0.4% CaCO_3_ pH 7.0); Sporulation medium (2% soluble starch and 0.4 yeast extract); and Oatmeal medium (2% oatmeal). These were cultivated at 28 °C and 220 rpm for 4 days in TSB; and for 8 days in SYP-NaCl, YD, SY, P1, P2, P3, CH-F2, Sporulation, and Oatmeal media. Once the bioprocess was completed, the broth was frozen at −20 °C for further chemical analysis.

### 2.7. Metabolic Profiling, Compound Isolation, and Chemical Structure Analysis

The *Streptomyces* broth of TSB, SYP-NaCl, and YD medium cultures (180 mL) was exhaustively extracted using a solvent mixture of 1:1 MeOH:DCM yielding a crude extract (3.89 g). This crude extract was first separated using SPE on C_18_ bonded silica gel (Polygoprep C18 (Fisher Scientific, Dublin, Ireland) 12%C, 60 Å, 40–63 µm), eluting with varying solvent mixtures to produce five fractions: H_2_O (743.62 mg), 1:1 H_2_O:MeOH (368.6 mg), MeOH (15.4 mg), 1:1 MeOH:DCM (10.9 mg), DCM (8.2 mg). The final three fractions (MeOH, 1:1 MeOH:DCM, DCM, 34.5 mg) were then combined and subject to analytical reverse phase HPLC on a Waters Symmetry (VWR, Dublin, Ireland) C18 5 µm, 4.6 × 250 mm column. The column was eluted with 10% MeCN (0.1% TFA)/90% H2O (0.1% TFA) for 5 min, then a linear gradient to 100% MeCN (0.1% TFA) over 21 min was performed. The column was further eluted with 100% MeCN (0.1% TFA) for 6 min. After the HPLC was complete, a linear gradient back to 10% MeCN (0.1% TFA)/90% H_2_O (0.1% TFA) over 1 min and then further elution of 10% MeCN (0.1% FA)/90% H_2_O (0.1% FA) for 4 min was performed. This yielded pure surugamide A (0.8 mg). Surugamide A was characterised using MS and NMR data to confirm the structure for use as an analytical standard.

Surugamide A was quantified in the broth using LC-MS analysis on an Agilent UHR-qTOF 6540 (Agilent Technologies, Cork, Ireland) mass spectrometer. The column used for separation was Waters equity UPLC BEH (Apex Scientific, Kildare, Ireland) C18 1.7 µm 2.1 × 75 mm. The column was eluted with 10% MeCN (0.1% FA)/90% H_2_O (0.1% FA) for 2 min, then a linear gradient to 100% MeCN (0.1% FA) over 6 min was performed. The column was further eluted with 100% MeCN (0.1% FA) for 4 min. After the UPLC was complete, a linear gradient back to 10% MeCN (0.1% FA)/90% H_2_O (0.1% FA) over 1 min and then further elution of 10% MeCN (0.1% FA)/90% H_2_O (0.1% FA) for 3 min was performed before the next run. The MS detection method was positive ion. A calibration curve was produce using the LC-MS method above and injecting the pure surugamide A at seven concentrations (100, 25, 10, 2, 1, 0.2, 0.1 mg/L). Thirty millilitres of each *Streptomyces* strain in broth were extracted using a solvent mixture of 1:1 MeOH:DCM three times to yield a crude extract. These extracts were resuspended in MeOH and filtered through PTFE 0.2 µm filters (Sigma Aldrich, Arklow, Ireland) before being subject to the above LC-MS method.

The surugamide A calibration standards 1–7 and the six extracts were analysed using the Agilent MassHunter Quantification software package. This allowed the quantification of surugamide A in the extracts based on the intensity of peaks in the chromatogram with matching retention time and exact mass.

## 3. Results and Discussion

### 3.1. Multi-locus Sequence Analysis and Taxonomy Assignment of the Streptomyces sp. SM17 Isolate

In order to taxonomically characterise the *Streptomyces* sp. SM17 isolate based on genetic evidence, multi-locus sequence analysis (MLSA) [51] employing the 16S rRNA sequence, in addition to five housekeeping genes, namely *atpD* (ATP synthase subunit beta), *gyrB* (DNA gyrase subunit B), *recA* (recombinase RecA), *rpoB* (DNA-directed RNA polymerase subunit beta), and *trpB* (tryptophan synthase beta chain) was performed, in a similar manner to a previous report [32]. A similarity search was performed in the GenBank database [35], using the NCBI BLASTN tool [36,37], based on the 16S rRNA nucleotide sequence of the SM17 isolate. The top 30 most similar *Streptomyces* species for which complete genome sequences were available in GenBank were selected for further phylogenetic analysis.

**Figure 1:**
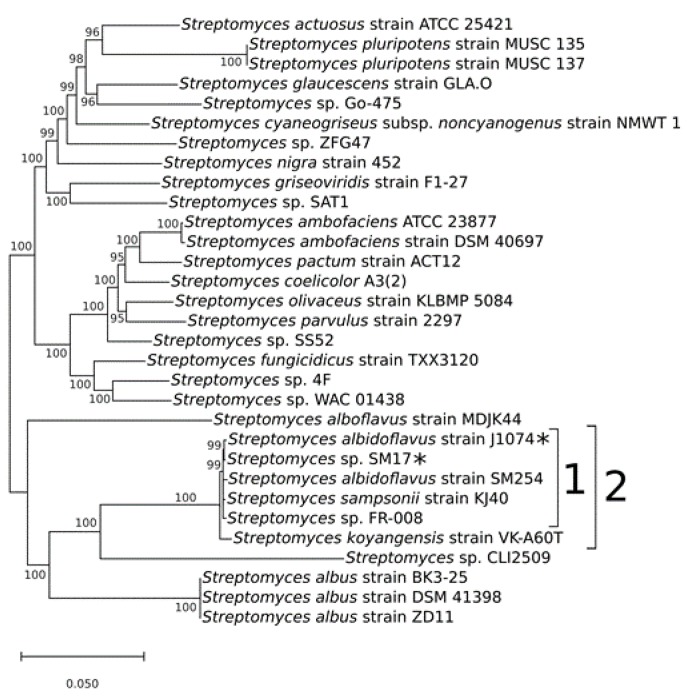
Phylogenetic tree of the concatenated sequences of the 16S rRNA and the housekeeping genes *atpD*, *gyrB*, *recA*, *rpoB*, and *trpB*, from the *Streptomyces* sp. SM17 together with 30 *Streptomyces* isolates for which complete genome sequences were available in the GenBank database. Analysis was performed using MrBayes, with a posterior probability cut-off of 95%. 1) *albidoflavus* phylogroup. 2) Clade including the neighbour isolate *Streptomyces koyangensis* strain VK-A60T. The strains SM17 and J1074 are indicated with asterisks.

The concatenated nucleotide sequences [51,52] of the 16S rRNA and the aforementioned five housekeeping genes, were first aligned using the MAFFT program [38], and the phylogeny analysis was performed using the MrBayes program [39]. The general time reversible (GTR) model of nucleotide substitution with gamma-distributed rates across sites with a proportion of invariable sites was applied [40], with 1 million generations sampled every 100 generations. The final phylogenetic tree was then processed using MEGA X [41], with a posterior probability cut-off of 95% (Figure 1).

The resulting phylogenetic tree clearly indicates the presence of a clade that includes the isolates *Streptomyces albidoflavus* strain J1074; *Streptomyces* sp. SM17; *Streptomyces albidoflavus* strain SM254; *Streptomyces sampsonii* strain KJ40; *Streptomyces* sp. FR-008; and *Streptomyces koyangensis* strain VK-A60T (clade 2 in Figure 1). In addition, this larger clade contains a sub-clade (clade 1 in Figure 1) that includes *Streptomyces* isolates similar to the strain *Streptomyces albidoflavus* J1074. The J1074 strain is a well-studied *Streptomyces* isolate widely used as a model for the genus and for various biotechnological applications, including the heterologous expression of secondary metabolites biosynthetic gene clusters (BGCs) [25,26,27,28,29]. This isolate was originally classified as “*Streptomyces albus* J1074”, but due to recent taxonomy data, it has been reclassified as *Streptomyces albidoflavus* J1074 [30,31]. Hence, in this study, this strain will be referred to as *Streptomyces albidoflavus* J1074, and this clade will from now on be referred to as the *albidoflavus* phylogroup (Figure 1).

Interestingly, members of the *albidoflavus* phylogroup were all isolated from quite different environments. The *Streptomyces albidoflavus* strain J1074 stems from the soil isolate *Streptomyces albus* G [23,24]. The *Streptomyces sampsonii* strain KJ40 was isolated from rhizosphere soil in a poplar plantation [53]. The *Streptomyces* sp. strain FR-008 is a random protoplast fusion derivative of two *Streptomyces hygroscopicus* isolates [54]. On the other hand, two of these strains were isolated from aquatic saline environments, with *Streptomyces* sp. SM17 being isolated from the marine sponge *Haliclona simulans* [13]; while the *Streptomyces albidoflavus* strain SM254 strain was isolated from copper-rich subsurface fluids within an iron mine, following growth on artificial sea water (ASW) [55]. The fact that these isolates, although derived from quite distinct environmental niches, simultaneously share significant genetic similarities is interesting, and raises questions about their potential evolutionary relatedness.

### 3.2. Analysis of Groups of Orthologous Genes in the Albidoflavus Phylogroup

In an attempt to provide further genetic evidence with respect to the similarities shared among the members of the *albidoflavus* phylogroup (Figure 1), a pan-genome analysis was performed to determine the number of core genes, accessory genes, and unique genes present in this group of isolates. The Roary program was employed for this objective [56], which allowed the identification of groups of orthologous and paralogous genes (which from now on will be referred to simply as “genes”) present in the set of *albidoflavus* genomes, with a protein identity cut-off of 95%, which is the identity value recommended by the Roary program manual when analysing organisms belonging to the same species.

**Figure 2:**
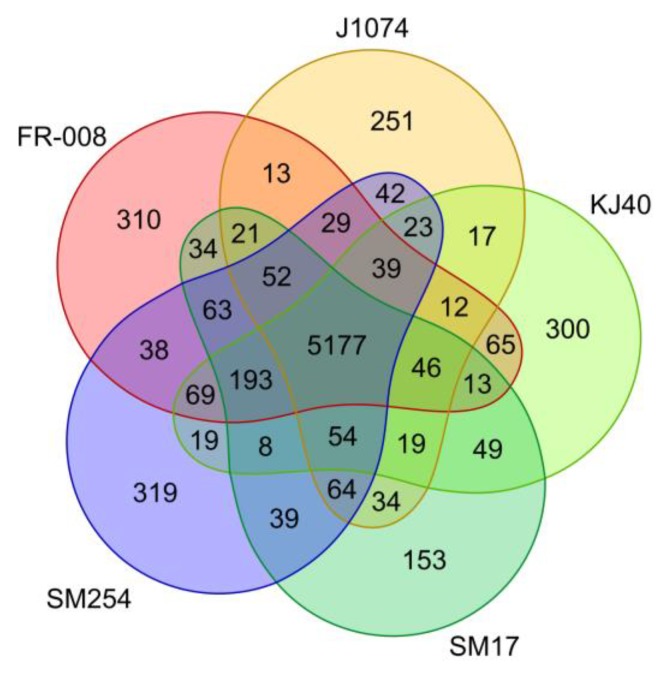
Venn diagram representing the presence/absence of groups of orthologous genes in the organisms belonging to the *albidoflavus* phylogroup.

A total of 7565 genes were identified in the *albidoflavus* pan-genome, and among these a total of 5177 were determined to be shared among all the *albidoflavus* isolates (i.e., the core genome) (Figure 2). This represents a remarkably high proportion of genes that appear to be highly conserved between all the isolates, representing approximately 68.4% of the pan-genome. Additionally, when considering the genomes individually (Table S1), the core genome accounts for approximately 84.5% of the FR-008 genome; 88.5% of J1074; 85.5% of KJ40; 86.7% of SM17; and 83.7% of the SM254 genome. On the other hand, the accessory genome (i.e., genes present in at least two isolates) was determined to consist of 1055 genes (or ~13.9% of the pan-genome); while the unique genome (i.e., genes present in only one isolate) was determined to consist of 1333 genes (or ~17.6% of the pan-genome). This strikingly high conservation of genes present in their genomes together with the previous multi-locus phylogeny analysis are very strong indicators that these microorganisms may belong to the same species.

An additional pan-genome analysis similar to the aforementioned analysis was also performed including the *Streptomyces koyangensis* strain VK-A60T in the dataset (Figure S1), which was an isolate shown to be a closely related neighbour to the *albidoflavus* phylogroup (Figure 1, clade 2). When compared to the previous analysis, the pan-genome analysis including the VK-A60T isolate showed significant changes in the values representing the core genome, which changed from 5177 genes (Figure 2) to 3912 genes (Figure S1), with an additional 1273 genes also shared among all of the *albidoflavus* isolates (Figure S1). The results also showed a much larger number of genes uniquely present in the VK-A60T genome than in the other genomes, with 2059 unique genes identified from a total of 6245 CDSs present in the VK-A60T genome in total, or approximately a third of its total number of genes (Figure S1). This proportion of unique genes present in the VK-A60T genome is considerably higher than the proportions of unique genes observed in the other *albidoflavus* phylotype genomes (Figure 2), which accounted for approximately only 2.5% of the total number of genes in SM17; 4.2% in J1074; 4.9% in KJ40; 5% in FR-008; and 5.1% in SM254. Taken together, these results further demonstrate the similarities between the isolates belonging to the *albidoflavus* phylogroup, while the VK-A60T isolate is clearly more distantly related.

Thus, from previous studies [30,31] and in light of the phylogeny analysis and further genomic evidence presented in this study, it is likely that all the isolates belonging to the *albidoflavus* phylogroup are in fact members of the same species. It is reasonable to infer that, for example, the isolates in the *albidoflavus* phylogroup that possess no species assignment thus far (i.e., strains SM17 and FR-008) are indeed members of the *albidoflavus* species. Also, it is possible that the *Streptomyces sampsonii* KJ40 has been misassigned, and possibly requires reclassification as an *albidoflavus* isolate.

Misassignment and reclassification of *Streptomyces* species is a common issue, and an increase in the quantity and the quality of available data from these organisms (e.g., better-quality genomes available in the databases) will provide better support for taxonomy claims, or correction of these when new information becomes available [31,57,58,59].

### 3.3. Prediction of Secondary Metabolites Biosynthetic Gene Clusters in the Albidoflavus Phylogroup

Isolates belonging to the *albidoflavus* phylogroup have been reported to produce bioactive compounds of pharmacological relevance, such as antibiotics. As mentioned previously, the *Streptomyces albidoflavus* strain J1074 is the best described member of the *albidoflavus* phylogroup to date. As such, several of secondary metabolites produced by this isolate have been identified, including acyl-surugamides and surugamides with antifungal and anticancer activities, respectively [16]; together with paulomycin derivatives with antibacterial activity [60]. The *Streptomyces* sp. FR-008 isolate has been shown to produce the antimicrobial compound FR-008/candicidin [61,62]; while the *Streptomyces sampsonii* KJ40 isolate has been shown to produce a chitinase that possesses anti-fungal activity against plant pathogens [53]. On the other hand, although no bioactive compounds have been characterised from *Streptomyces albidoflavus* SM254, this isolate has been shown to possess anti-fungal activity, specifically against the fungal bat pathogen *Pseudogymnoascus destructans*, which is responsible for the White-nose Syndrome [55,63]. The *Streptomyces* sp. SM17 isolate has also previously been shown to possess antibacterial and antifungal activities against clinically relevant pathogens, including methicillin-resistant *Staphylococcus aureus* (MRSA) [13]. However, no natural products derived from this strain have been identified and isolated until now.

**Figure 3:**
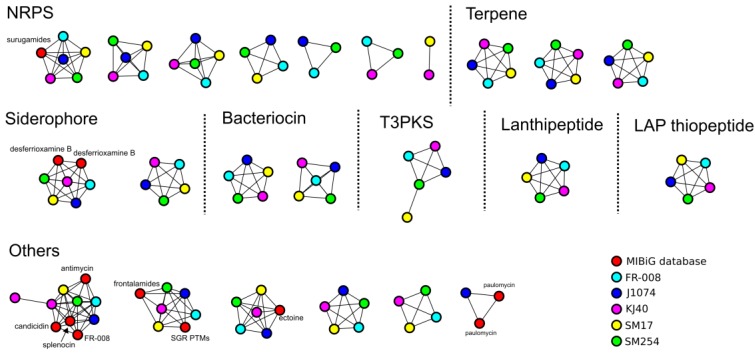
Biosynthetic gene clusters (BGCs) similarity clustering using BiG-SCAPE. Singletons, i.e., BGCs without significant similarity with the BGCs from the minimum information about a biosynthetic gene cluster (MiBIG) database or with the BGCs predicted in other genomes, are not represented.

**Figure 4:**
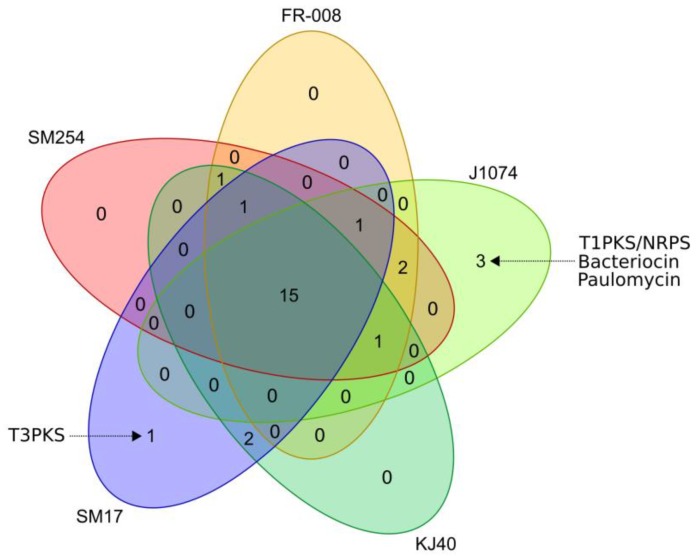
Venn diagram representing BGCs presence/absence in the genomes of the members of the *albidoflavus* phylogroup, determined using antiSMASH and BiG-SCAPE.

In order to further *in silico* assess the potential of these *albidoflavus* phylogroup isolates to produce secondary metabolites, and also to determine how potentially similar or diverse they are within this phylogroup, prediction of secondary metabolites biosynthetic gene clusters (BGCs) was performed using the antiSMASH (version 5) program [42]. The antiSMASH prediction was processed using the BiG-SCAPE program [43], in order to cluster the BGCs into gene cluster families (GCFs), based on sequence and Pfam [64] protein families similarity, and also by comparing them to the BGCs available from the minimum information about a biosynthetic gene cluster (MiBIG) repository [44] (Figure 3). When compared to known BGCs from the MiBIG database, a significant number of BGCs predicted to be present in the *albidoflavus* phylogroup genomes could potentially encode for the production of novel compounds, including those belonging to the non-ribosomal peptide synthetase (NRPS) and bacteriocin families of compounds (Figure 3). The presence/absence of homologous BGCs in the *albidoflavus* isolates’ genomes was determined using BiG-SCAPE and is represented in Figure 4. Interestingly, the vast majority of the BGCs predicted in the *albidoflavus* phylogroup are shared among all of its members (15 BGCs); while another large portion (8 BGCs) are present in at least two isolates (Figure 4). Among the five members of the *albidoflavus* phylogroup, only the J1074 strain and the SM17 strain appeared to possess unique BGCs when compared to the other strains. Three unique BGCs were predicted to be present in the J1074 genome: a predicted type I polyketide synthase (T1PKS)/NRPS without significant similarity to the BGCs from the MiBIG database; a predicted bacteriocin, which also did not show any significant similarity to the BGCs from the MiBIG database; and a BGC predicted to encode for the production of the antibiotic paulomycin, with similarity to the paulomycin-encoding BGCs from *Streptomyces paulus* and *Streptomyces* sp. YN86 [65], which has also been experimentally shown to be produced by the J1074 strain [60]. One BGC predicted to encode a type III polyketide synthase (T3PKS)—with no significant similarity to the BGCs from the MiBIG database—was also identified as being unique to the SM17 genome.

Importantly, BGCs with similarity to the surugamide A/D BGC from “*Streptomyces albus* J1074” (now classified as *S. albidoflavus*) from the MiBIG database [16] were identified in all the other genomes of the members of the *albidoflavus* phylogroup. This raises the possibility that this BGC may be commonly present in *albidoflavus* species isolates. However, as only a few complete genomes of isolates belonging to this phylogroup are currently available, further data will be required to support this hypothesis. Nevertheless, these results further highlight the genetic similarities of the isolates belonging to the *albidoflavus* phylogroup, even with respect to their potential to produce secondary metabolites.

### 3.4. Phylogeny and Gene Synteny Analysis of Sur BGC Homologs

In parallel to the previous phylogenomics analysis performed with the *albidoflavus* phylogroup isolates, sequence similarity and phylogenetic analyses were performed, using the previously described and experimentally characterised *Streptomyces albidoflavus* LHW3101 surugamides biosynthetic gene cluster (*sur* BGC, GenBank accession number: MH070261) as a reference [18]. The aim was to assess how widespread in nature the *sur* BGC might be, and the degree of genetic variation, if any; that might be present in *sur* BGCs belonging to different microorganisms.

Nucleotides sequence similarity to the *sur* BGC was performed in the GenBank database [35], using the NCBI BLASTN tool [36,37]. It is important to note that, since the quality of the data is crucial for sequence similarity, homology, and phylogeny inquiries, only complete genome sequences were employed in this analysis. For this reason, for example, the marine *Streptomyces* isolate in which surugamides and derivatives were originally identified, namely *Streptomyces* sp. JAMM992 [14], was not included, since its complete genome is not available in the GenBank database.

The sequence similarity analysis identified five microorganisms that possessed homologs to the *sur* BGC and had their complete genome sequences available in the GenBank database: *Streptomyces* sp. SM17; *Streptomyces albidoflavus* SM254; *Streptomyces* sp. FR-008; *Streptomyces albidoflavus* J1074; and *Streptomyces sampsonii* KJ40. Notably, these results overlapped with the isolates belonging to the previously discussed *albidoflavus* phylogroup (Figure 1), further highlighting the possibility that the *sur* BGC may be commonly present in and potentially exclusive to the *albidoflavus* species.

**Figure 5:**
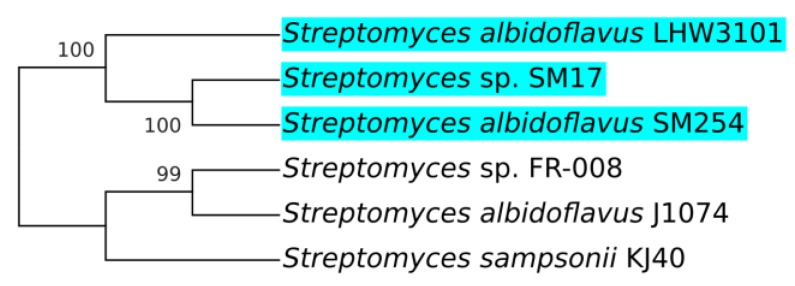
Consensus phylogenetic tree of the *sur* BGC region of the *S. albidoflavus* LHW3101 reference *sur* BGC sequence, plus five *Streptomyces* isolates determined to have *sur* BGC homologs, generated using MrBayes and Mega X, with a 95% posterior probability cut-off. Aquatic saline environment-derived isolates are highlighted in cyan.

Phylogenetic analysis was performed in the genomic regions determined to be homologs to the *Streptomyces albidoflavus* LHW3101 *sur* BGC, using the MrBayes program [39] (Figure 5). Although a larger number of sequences should ideally be employed in this type of analysis, these results suggest the possibility of a clade with aquatic saline environment-derived *sur* BGCs (Figure 5). Thus, these aquatic saline environment-derived *sur* BGCs are likely to share more genetic similarities amongst each other, rather than with those derived from terrestrial environments. Since this analysis took into consideration the whole genome regions that contained the *sur* BGCs of each isolate, it is likely that the similarities and differences present in these regions involve not only coding sequences (CDSs) for biosynthetic genes and/or transcriptional regulators, but also could include promoter regions and other intergenic sequences.

**Figure 6:**
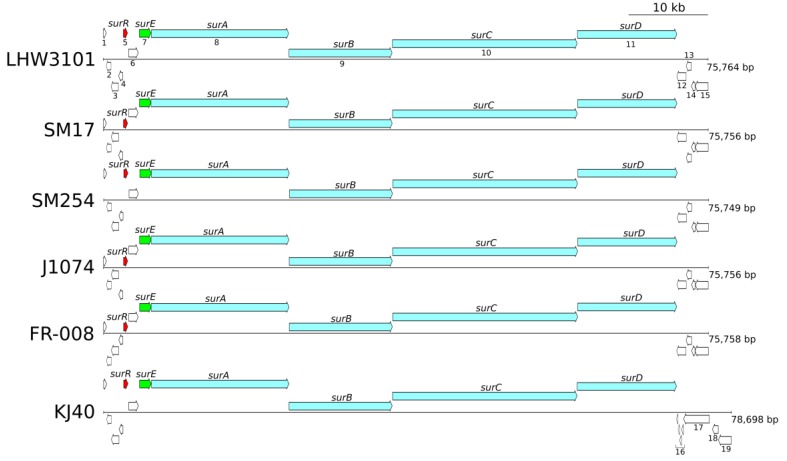
Gene synteny of the *sur* BGC region, including the reference *sur* BGC nucleotide sequence (LHW3101) and each of the *albidoflavus* phylogroup genomes. Arrows at different positions represent genes transcribed in different reading frames.

With this in mind, the genomic regions previously determined to share homology with the *sur* BGC from *S. albidoflavus* LHW3101 were further analysed, with respect to the genes present in the surrounding region, the organisation of the BGCs, together with the overall gene synteny (Figure 6). Translated CDSs predicted in the region were manually annotated using the NCBI BLASTP tool [36,37], together with GenBank [35] and the CDD [66] databases. These included the main biosynthetic genes, namely *surABCD*, the transcriptional regulator *surR*, and the thioesterase *surE*—all of which had previously been reported to have roles in the biosynthesis of surugamides and their derivatives [15,16,17,18,19] (Figure 6).

Interestingly, this result indicated that the gene synteny of the biosynthetic genes as well as the flanking genes is highly conserved, with the exception to the 3’ flanking region of the BGC from *S. sampsonii* KJ40. Notably, even the reading frames of the *surE* gene and the *surABCD* genes are conserved amongst all the genomes. As indicated by the numbers in Figure 6, the 5’ region in all the genomic regions consisted of: 1) A MbtH-like protein, which have been reported to be involved in the synthesis of non-ribosomal peptides, antibiotics, and siderophores, in *Streptomyces* species [67,68]; 2) a putative ABC transporter, which is a family of proteins with varied biological functions, including conferring resistance to drugs and other toxic compounds [69,70]; 3) a BcrA family ABC transporter, which is a family commonly involved in peptide antibiotics resistance [71,72]; 4) a hypothetical protein; followed by 5) the transcriptional repressor SurR, which has been experimentally demonstrated to repress the production of surugamides [16]; 6) a hypothetical membrane protein; 7) the thioesterase SurE, which is homologous to the penicillin binding protein, reported to be responsible for the cyclisation of surugamides molecules [21]; and finally 8–11) the main surugamides biosynthetic genes *surABCD*, all of which encode non-ribosomal peptide synthetase (NRPS) proteins [19]. The 3’ flanking region consisted of: 12) A predicted multi-drug resistance (MDR) transporter belonging to the major facilitator superfamily (MFS) of membrane transport proteins [73,74]; 13) a predicted TetR/AcrR transcriptional regulator, which is a family of regulators reported to be involved in antibiotic resistance [75]; 14) a hypothetical protein; and 15) another predicted MDR transporter belonging to the MFS superfamily. In contrast, the 3’ flanking region of the KJ40 strain *sur* BGC, consisted of: 16) A group of four hypothetical proteins, which may represent pseudogene versions of the first MDR transporter identified in the other isolates (gene number 12 in Figure 6); 17) a predicted rearrangement hotspot (RHS) repeat protein, which is a family of proteins reported to be involved in mediating intercellular competition in bacteria [76]; 18) a hypothetical protein; and 19) a MDR transporter belonging to the MFS superfamily, which, interestingly, is a homolog of protein number 15, which is present in all the other isolates.

The conserved gene synteny observed in the *sur* BGC genomic region, particularly those positioned upstream of the main biosynthetic *surABCD* genes, together with the observation that even the reading frames of the *surE* and the *surABCD* genes are conserved among all the genomes analysed, coupled with the previous phylogenetic and pan-genome analyses, suggest the following. Firstly, it is very likely that these strains share a common ancestry and that the *sur* BGC genes had a common origin. Secondly, there is a strong evolutionary pressure ensuring the maintenance of not only gene synteny, but also of the reading frames of the main biosynthetic genes involved in the production of surugamides. The latter raises the question of which other genes in this region may be involved in the production of these compounds, or potentially conferring mechanisms of self-resistance to surugamides in the isolates, particularly since many of the genes have predicted functions that are compatible with the transport of small molecules and with multi-drug resistance. These observations are particularly interesting considering that these strains are derived from quite varied environments and geographic locations.

### 3.5. Growth, Morphology, Phenotype, and Metabolism Assessment of Streptomyces sp. SM17 in Complex Media

In order to assess the metabolic potential of the SM17 strain [77], particularly with respect to the production of surugamide A, the isolate was cultivated in a number of different growth media, within an OSMAC-based approach [33,34]. While the SM17 strain was able to grow in SYP-NaCl, YD, SY, P1, P2, P3, and CH-F2 liquid media, the strain was unable to grow in Oatmeal and Sporulation media. The latter indicated an inability to metabolise oat and starch when nutrients other than yeast extract are not present. Morphologically, the SM17 strain formed cell aggregates or pellets in TSB, YD, and SYP-NaCl, while this differentiation was not observed in the other media. Preliminary chemical analyses of these samples, employing liquid chromatography–mass spectrometry (UPLC-DAD-HRMS), indicated that secondary metabolism in SM17 was not very active when the strain was cultivated in SY, P1, P2, P3, and CH-F2 media. In contrast, significant production of surugamides was evidenced in the extracts from TSB, SYP-NaCl, and YD media, with characteristic ions at *m/z* 934.6106 (surugamide A) and 920.5949 (surugamide B) [M + Na]^+^, which correlated with the formation of cell pellets and the production of natural products, as previously described in other *Streptomyces* strains [77,78].

### 3.6. Differential Production of Surugamide A by Streptomyces sp. SM17 and S. albidoflavus J1074

**Figure 7:**
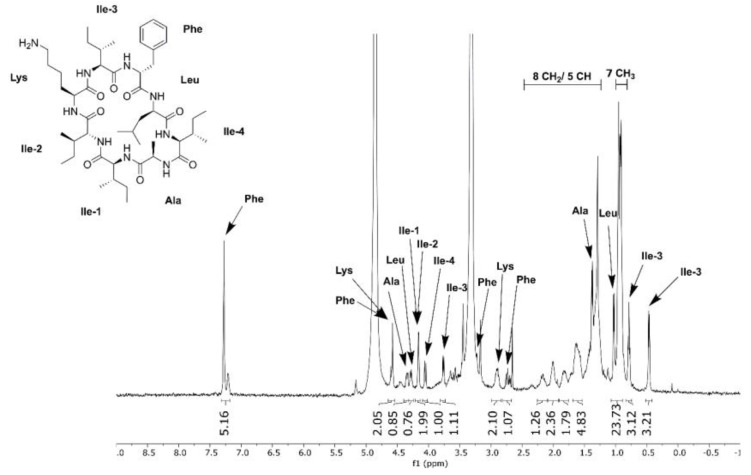
Structure of surugamide A isolated from SM17 grown in TSB, SYP-NaCl, and YD medium with annotated ^1^H NMR spectrum obtained in CD_3_OD at 500 MHz.

To confirm the production of surugamide A by the SM17 isolate, extracts from the TSB, SYP-NaCl, and YD media were combined and purified using high-performance liquid chromatography (HPLC). The structures of the major compounds of the extract were subsequently analysed using nuclear magnetic resonance (NMR) spectroscopy, which allowed for the identification of the chemical structure of the surugamide A molecule as major metabolite by comparison with reference NMR data (Figure 7) [14].

The isolates *Streptomyces* sp. SM17 and *S. albidoflavus* J1074 were subsequently cultivated in the aforementioned media in which the SM17 strain had been shown to be metabolically active, namely the TSB, SYP-NaCl, and YD media. This was performed in order to assess whether there were any significant differences in the production of surugamide A when different growth media are employed for the production of this compound, and to compare the levels of surugamide A produced by the SM17 and the J1074 isolates. The MeOH/DCM (1:1) extracts from the aforementioned cultures of SM17 and J1074 were subjected to liquid chromatography–mass spectrometry (UPLC-HRMS) to quantify the levels of surugamide A being produced under each condition (Table 1), using a surugamide A standard calibration curve (Figure S2).

The LC-MS quantification analysis (Table 1) indicated that both strains were capable of producing surugamide A in all the conditions tested. However, the SM17 strain appeared to produce considerably higher yields of the compound when compared to J1074, in all the conditions analysed. In addition, the *S. albidoflavus* J1074 isolate appeared to produce quite low levels of surugamide A when grown in TSB and YD media, accounting for less than 1% (*w*/*w*) of the extracts from these media. Interestingly, higher yields of surugamide A were produced in the SYP-NaCl medium in both strains, when compared with the levels of surugamide A produced by these strains when grown in TSB and the YD media (Table 1). In the SM17 culture in SYP-NaCl, surugamide A accounted for 10.60% (*w*/*w*) of the extract, compared to 2.44% and 1.13% from TSB and YD, respectively; while in J1074 it accounted for 3.55% (*w*/*w*) of the extract from the SYP-NaCl culture, compared to 0.27% and 0.09% from TSB and YD, respectively (Table 1). These results provide further insights into factors that are potentially involved in regulation the biosynthesis of surugamide A, in the *albidoflavus* phylogroup and in *Streptomyces* sp. SM17 in particular.

Firstly, it appears likely that surugamide A biosynthesis may be regulated, at least in part, by carbon catabolite repression (CCR). Carbon catabolite repression is a well-described regulatory mechanism in bacteria that controls carbon metabolism [79,80,81,82], and which has also been reported to regulate the biosynthesis of secondary metabolites in a number of different bacterial species, including in *Streptomyces* isolates [83,84,85,86]. While the TSB and the YD media contain glucose and dextrins as carbon sources, respectively; the complex polysaccharide starch is the carbon source in the SYP-NaCl medium. Therefore, it is reasonable to infer that glucose and dextrin may repress the production of surugamide A in *Streptomyces* sp. SM17 and in *Streptomyces albidoflavus* J1074, while starch does not. Further evidence for this can be found when considering the different production media previously employed in the production of surugamides by different *Streptomyces* isolates. For example, in the original research that led to the discovery of surugamides in *Streptomyces* sp. JAMM992 [14], the PC-1 medium (1% starch, 1% polypeptone, 1% meat extract, 1% molasses, pH 7.2) was employed for production of these compounds. Similar to the SYP-NaCl medium employed in our study, the PC-1 medium also contains starch as the carbon source, together with another complex carbon source, namely molasses. Likewise, for the production of surugamides in *S. albidoflavus* strain LHW3101 [18], the TSBY medium (3% tryptone soy broth, 10.3% sucrose, 0.5% yeast extract) was employed, which utilises sucrose as its main carbon source. In contrast, when elicitors were employed to induce the production of surugamides and their derivatives in the J1074 strain [16], by activating the *sur* BGC, which appeared to be silent in this isolate, the R4 medium (0.5% glucose, 0.1% yeast extract, among other non-carbon related components) was employed, which utilises glucose as its main carbon supply, and, as shown in this study, it potentially represses the production of surugamide A. Thus, from these previous reports and from our observations, it appears likely that CCR plays an important role in regulating the biosynthesis of surugamides.

Secondly, it is important to note the presence of salts in the form of NaCl in the SYP-NaCl medium. As previously mentioned, genetic and phylogenetic analyses of the *sur* BGC indicated similarities between those BGCs belonging to aquatic saline-derived *Streptomyces* isolates (Figure 5), together with the likelihood that these *sur* BGCs might have had a common origin. Thus, it is plausible that this origin may have been marine, and hence the presence of salts in the growth medium may also have an influence on the biosynthesis of surugamide A. Different concentrations of salts in the form of NaCl in the culture medium have also previously been shown to impact on the chemical profile of metabolites produced in the marine-obligate bacteria *Salinispora arenicola* [87].

Nevertheless, it is interesting to observe that, despite the repression/induction of the biosynthesis of surugamide A observed when different media were employed, the SM17 isolate clearly produces considerably higher amounts of surugamide A when compared to *S. albidoflavus* J1074—reaching yields up to >13-fold higher in the YD medium, and around 3-fold higher when grown in the SYP-NaCl medium (Table 1).

## 4. Conclusions

Marine-derived bacteria, particularly those isolated in association with marine invertebrates, such as sponges, have been shown to be reservoirs of bioactive molecules, including those with antibacterial, antifungal, and anticancer activities. Among these newly identified bioactive compounds, the surugamides and their derivatives are of particular interest due to their clinically relevant bioactivities, i.e., anticancer and antifungal, and their original metabolic pathway.

Based on genome mining, this study identified the previously unreported capability of the marine sponge-derived isolate *Streptomyces* sp. SM17 to produce surugamide A and also sheds new light on factors such as the carbon catabolite repression (CCR) that may be involved in regulating production of this molecule. Phylogenomics analysis indicated that the *sur* BGC is commonly present in members of the proposed *albidoflavus* phylogroup, and that the *sur* BGCs present in different isolates derived from varied environmental niches may possess a common ancestry. Although high quality genomic data from this proposed *albidoflavus* phylogroup are still lacking, results presented here suggest that the *sur* BGCs derived from *Streptomyces* isolated from aquatic saline environment are more similar to each other, when compared to those isolated from terrestrial environments.

Chemical analysis was performed in order to assess differential production of surugamide A when comparing a marine *Streptomyces* isolate with a terrestrial *Streptomyces* isolate, namely SM17 and J1074 strains, respectively, following an OSMAC-based approach employing different culture media. This analysis showed that not only the marine-derived isolate SM17 was capable of producing more surugamide A when compared to J1074 under all the conditions tested, but also that the biosynthesis of surugamide A is likely to be influenced by the CCR, and potentially by the presence of salts in the growth medium. These results also highlight the importance of employing an OSMAC-based approach even when analysing the production of known compounds, since there is a clear difference in the yields of surugamide A obtained when employing different culture media. Thus, it is possible to gain further insights into the production of bacterial types of compounds by 1) discovering strains that possess a higher capability to produce these compounds; 2) establishing optimal conditions for the biosynthesis of their production; and 3) providing a better understanding of the genetic and regulatory mechanisms potentially underpinning the production of these compounds.

## Figures and Tables

**Table 1:** Surugamide A production by SM17 and J1074 measured using different media.

Strain	Media	Percent (*w*/*w*) of Extract	Concentration of Surugamide A (mg/L) Corrected in 5 mg/mL of Extract
SM17	TSB	2.44%	122.01
SM17	SYP-NaCl	10.60%	530.15
SM17	YD	1.13%	56.27
J1074	TSB	0.27%	13.32
J1074	SYP-NaCl	3.55%	176.82
J1074	YD	0.09%	4.26

## Supplementary Materials

The following are available online at https://www.mdpi.com/2076-2607/7/10/394/s1, Figure S1: Groups of orthologous genes in the genomes of the *albidoflavus* phylogroup, Figure S2: Calibration curve for surugamide A, Table S1: Genome statistics of the *albidoflavus* phylogroup.
