# Supplementary material for: Genome Mining Coupled with OSMAC-Based Cultivation Reveal Differential Production of Surugamide A by the Marine Sponge Isolate Streptomyces sp. SM17 When Compared to Its Terrestrial Relative S. albidoflavus J1074
Source: Microorganisms. 2019 Sep 26;7(10):394. doi: 10.3390/microorganisms7100394 (PMC6843307; doi:10.3390/microorganisms7100394)
Supplement: Supplementary file 1 [file microorganisms-07-00394-s001.pdf]

Supplementary Tables and Figures

Table S1. Genome statistics determined using the Prokka program, of the *Streptomyces* isolates genomes obtained from GenBank and determined to belong to the *albidoflavus* phylogroup.

Isolate	Number of bases	Number of CDSs	rRNA	tRNA	tmRNA
FR-008	7,090,955	6,126	21	79	1
J1074	6,841,649	5,847	21	77	1
KJ40	7,070,328	6,057	21	78	1
SM17	6,975,788	5,972	21	78	1
SM254	7,170,504	6,182	21	77	1

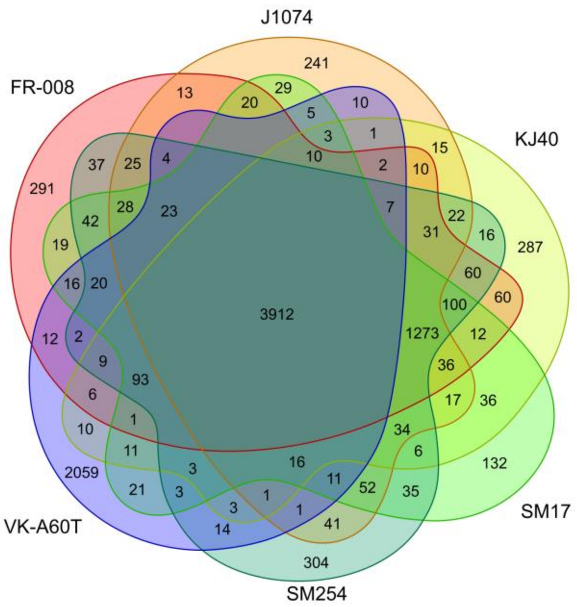

Figure S1. Venn diagram representing the presence/absence of groups of orthologous genes in the *albidoflavus* phylogroup genomes (namely strains FR-008, J1074, KJ40, SM17, and SM254), also including the *Streptomyces koyangensis* VK-A60T genome.

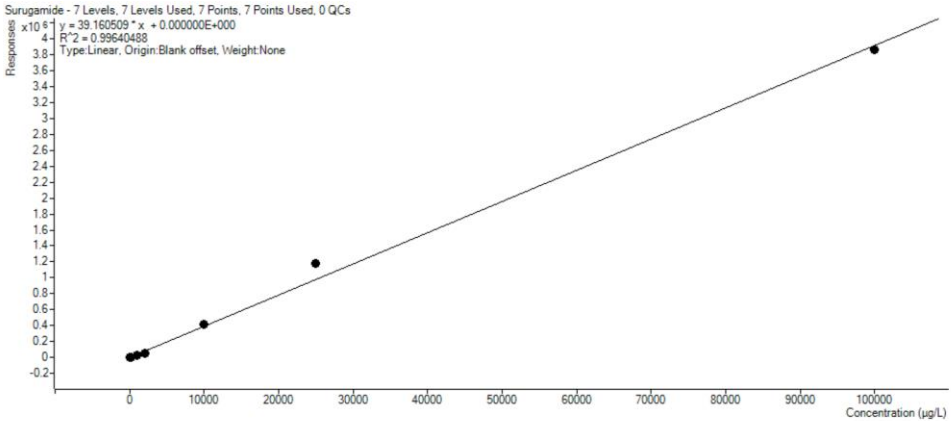

Figure S2. Calibration curve for surugamide A, determined using LC-MS and pure surugamide A at seven concentrations (0.1, 0.2, 1, 2, 10, 25, 100 mg/L).
